# 5-Aminosalicylic Acid Inhibits Acute *Clostridium difficile* Toxin A-Induced Colitis in Rats

**DOI:** 10.1155/2014/389621

**Published:** 2014-06-23

**Authors:** Steven R. Vigna

**Affiliations:** ^1^Departments of Cell Biology & Medicine, Duke University Medical Center, P.O. Box 3011, Durham, NC 72210, USA; ^2^VA Medical Center, Durham, NC 27705, USA

## Abstract

We tested the hypothesis that 5-aminosalicylic acid (5-ASA) inhibits toxin A-induced generation of colonic leukotriene B_4_ (LTB_4_) and toxin A colitis in rats. Isolated colonic segments in anesthetized rats were treated intraluminally with toxin A for 3 hours with or without 30 minutes of pretreatment with either 5-ASA or sulfapyridine and then colonic tissue levels of LTB_4_ were measured and inflammation was assessed. Separately, sulfasalazine was administered to rats in their drinking water for 5 days, isolated colonic segments were then prepared, toxin A was administered, and inflammation was assessed as before. Pretreatment with 5-ASA inhibited toxin A-induced increased tissue LTB_4_ concentration in the colon. Sulfasalazine and 5-ASA but not sulfapyridine significantly inhibited toxin A colitis. However, pretreatment with 5-ASA did not protect against direct TRPV1-mediated colitis caused by capsaicin. Toxin A stimulated the release of substance P (SP), and this effect was also inhibited by sulfasalazine and 5-ASA but not by sulfapyridine. Thus, toxin A stimulates colonic LTB_4_ resulting in activation of TRPV1, release of SP, and colitis. Inhibition of 5-LO by 5-ASA disrupts this pathway and supports the concept that LTB_4_ activation of TRPV1 plays a role in toxin A colitis.

## 1. Introduction

*Clostridium difficile* produces exotoxins such as toxin A that cause acute colonic inflammation characterized clinically by watery diarrhea and cramps and pathologically by pseudomembranous colitis. The pathway by which toxin A causes colitis is incompletely known. We have shown that oral sulfasalazine inhibits toxin A colitis in rats [1]. Sulfasalazine is used to treat the chronic human inflammatory bowel diseases (IBDs), ulcerative colitis (UC), and Crohn's disease (CD). Sulfasalazine consists of one molecule of 5-aminosalicylic acid (5-ASA, mesalamine) coupled by an azo bond to one molecule of sulfapyridine. The azo bond allows uncoupling of the two constituent compounds in the lumen of the colon by the action of bacterial azo reductase enzymes resulting in topical delivery of the compounds [2]. It has been shown that the 5-ASA moiety of sulfasalazine is the therapeutically active component in UC and CD [3, 4] and that the sulfapyridine moiety is inactive and causes most of the allergic and intolerant effects of sulfasalazine [5, 6].

The mechanism of the therapeutic effect of 5-ASA in intestinal inflammation is unknown. Several possible mechanisms have been proposed including inhibition of 5-lipoxygenase (5-LO), the rate-limiting enzyme in the biosynthesis of leukotriene B_4_ (LTB_4_) [5, 7]. Inhibition of 5-LO is a promising candidate because of the demonstrated relationship between LTB_4_ and human UC [5] and because of the efficacy of other 5-LO inhibitors in UC [8]. LTB_4_ is also found in much higher concentrations in patients with IBD than in healthy controls [5]. In addition, LTB_4_ has been shown to be increased in animal models of colitis [9–12] and inhibition of 5-LO caused reduction of tissue LTB_4_ levels and inhibited colitis in these models [11, 13].

We have shown previously that LTB_4_ activates the transient receptor potential vanilloid-1 (TRPV1) ion channel expressed by primary sensory neurons in the ileum resulting in neurogenic inflammation and that inhibition of TRPV1 reduces LTB_4_- and toxin A-induced ileitis [14, 15]. The fact that LTB_4_ is an endogenous TRPV1 agonist [16] coupled with our demonstration that inhibition of 5-LO inhibited both toxin A-induced ileal LTB_4_ levels and toxin A- but not LTB_4_-induced ileitis led us to propose that* C. difficile* toxin A causes ileitis by stimulating mucosal LTB_4_ production that subsequently initiates neurogenic inflammation mediated by TRPV1.

We show here that 5-ASA decreases toxin A-stimulated colonic LTB_4_ levels and SP release and that 5-ASA but not sulfapyridine inhibits toxin A-induced colitis. However, 5-ASA does not inhibit direct TRPV1-mediated colitis caused by intraluminal capsaicin. Taken together, these results demonstrate that 5-ASA inhibits 5-LO and accounts for the protective effects of sulfasalazine in toxin A colitis.

## 2. Materials and Methods

### 2.1. Materials

Sulfasalazine, 5-aminosalicylic acid (5-ASA, mesalamine), sulfapyridine, and capsaicin were purchased from Sigma (St. Louis, MO). 5′-Iodoresiniferatoxin (I-RTX) was purchased from Tocris Cookson (Ellisville, MO).* Clostridium difficile* toxin A was purchased from TechLab, Inc. (Blacksburg, VA).

### 2.2. Surgery

Isolated colonic segments were constructed in anesthetized male Sprague-Dawley rats (150–175 g) as previously described for construction of ileal segments [17, 18]. Isolated colonic segments 5 cm in length were constructed distal to the caecum by ligation with silk sutures.

### 2.3. Drug Administration

Toxin A was administered at a dose of 5 *μ*g in 400 *μ*L (or 200 *μ*L when given after other drugs) of PBS into the lumen of the isolated colonic segments. 5-ASA was dissolved in phosphate buffered saline (PBS) and injected intraluminally in volumes of 200 *μ*L 30 minutes prior to toxin A injection. Sulfapyridine was initially dissolved in dimethyl sulfoxide (DMSO) and then diluted to 10% DMSO in PBS. Capsaicin was administered at a dose of 4 mg in 400 *μ*L (or 200 *μ*L when given after other drugs) of 25% ethanol in saline. All intraluminal injections were made using a 27 ga syringe needle. Control rats were prepared identically and their isolated colonic segments were injected with the appropriate vehicle solutions. Sulfasalazine was initially dissolved in 0.1  N NaOH at 30 times the desired final concentration and then the pH was adjusted to 8.5 with 1 N HCl and the solution was diluted 30-fold with distilled water. Sulfasalazine was administered chronically in this solution as drinking water for 5 days. The final concentration of sulfasalazine used was calculated from preliminary data obtained on the amount of water that the animals drank per day. The drinking water for control animals was prepared identically with the omission of sulfasalazine. The animals drank the same amount of the sulfasalazine vehicle as tap water. These studies were approved by the Duke University and Durham VA Institutional Animal Care and Use Committees.

### 2.4. Luminal Fluid Accumulation

Luminal fluid accumulation was measured gravimetrically. After 3 hours of treatment, the isolated colonic segments were removed and weighed, and their lengths were measured. Luminal fluid accumulation is expressed as mg wet weight per cm length.

### 2.5. Myeloperoxidase Activity

Myeloperoxidase (MPO) activity was measured as described previously [19]. Briefly, pieces of control and treated colonic segments were homogenized in 0.5% hexadecyltrimethylammonium bromide in 50 mM KH_2_PO_4_ (pH 6), freeze-thawed three times, and centrifuged at 4°C for 2 minutes, and then the absorbance of each supernatant was read at 460 nm at 0, 30, and 60 seconds after the addition of 2.9 mL of* o*-dianisidine dihydrochloride to 0.1 mL supernatant. The maximal change in absorbance per minute was used to calculate the units of MPO activity based on the molar absorbency index of oxidized* o*-dianisidine of 1.13 × 10^4^ M^−1^ cm^−1^. The results are expressed as MPO units of activity per gram of tissue wet weight.

### 2.6. Histopathology

After 3 hours of treatment, equivalent portions of the isolated colonic segments were fixed overnight in 10% formalin, dehydrated, and embedded in paraffin, and then sections of 5 *μ*m in thickness were cut, mounted on glass slides, and stained with hematoxylin and eosin.

### 2.7. LTB_4_ Measurement

Colonic LTB_4_ levels were measured by LTB_4_ enzyme immunoassay (EIA) kits purchased from Cayman Chemical (Ann Arbor, MI) as previously described [15]. Briefly, samples of colon and colonic luminal fluid were collected after various treatments in 5 volumes of ice-cold 0.1 M phosphate buffer, pH 7.4, containing 1 mM EDTA and 10 *μ*M indomethacin and homogenized for 15 sec on ice using a Tekmar Tissumizer (Tekmar, Cincinnati, OH) at a 50% power setting. Before homogenization, 10,000 cpm of ^3^H-LTB_4_ (120–240 Ci/mmol; Perkin-Elmer Life Sciences, Boston, MA) was added to the buffer for later assessment of LTB_4_ recovery. After homogenization, 2 volumes of ice-cold ethanol were added to each extract and the extracts were then incubated on ice for 5 min to precipitate proteins. After centrifugation at 3000 ×g_max⁡_ to remove the precipitated proteins, the ethanol in the supernatants was removed by vacuum centrifugation. The pH of the extracts was adjusted to ~4.0 by addition of 1 M sodium acetate (pH 4.0). The resulting precipitate was removed by centrifugation and the supernatant was loaded onto C-18 solid phase extraction cartridges (Cayman Chemical, Ann Arbor, MI) previously washed with methanol and distilled water, washed with distilled water followed by hexane, and then eluted at unit gravity with 5 mL of 99% ethyl acetate: 1% methanol. The samples were then evaporated to dryness by vacuum centrifugation, reconstituted in LTB_4_ EIA buffer, and assayed according to the instructions of the kit manufacturer. Because toxin A caused portions of the colonic mucosa to slough off into the intestinal lumen, luminal contents were collected by syringe from toxin A-treated colonic segments and assayed for LTB_4_ content just as for colonic tissue, and the LTB_4_ contents of the colonic tissue and corresponding luminal contents were added together for these samples. Because it seemed inappropriate to express the results as LTB_4_ concentrations per unit wet weight, therefore, the results are expressed as LTB_4_ concentrations per cm of colonic length.

### 2.8. Substance P Release

Substance P (SP) release was assessed by analysis of NK-1R endocytosis as described previously [14, 20] with modifications. Briefly, pieces of colonic segments taken from control, toxin A-treated, and capsazepine pretreated/toxin A-treated rats were fixed in freshly depolymerized 4% paraformaldehyde overnight at 4°C. The tissue was then washed and embedded in Tissue Tek O.C.T. compound (Sakura, Torrance, CA), frozen, sectioned at 20 *μ*m, and mounted on Superfrost/Plus glass slides (Fisher, Pittsburgh, PA). After washing, the slides were stained using a rabbit antiserum (number 11886-5) specific for the C-terminal 15 amino acids of the rat NK-1R at a dilution of 1 : 3000 [21]. This was followed by incubation in a cyanine 3-conjugated donkey anti-rabbit IgG secondary antibody (Jackson ImmunoResearch, West Grove, PA) at a dilution of 1 : 600. The stained sections were analyzed using a Zeiss LSM-510 META inverted krypton-argon confocal laser scanning system coupled to a Zeiss Axiovert 200 MOT microscope. Images of 512 × 512 pixels were obtained and processed using Adobe Photoshop. Quantification of NK-1R endocytosis was achieved by analyzing 20 NK-1R-immunoreactive (NK-1R-ir) myenteric plexus neuronal cell bodies per rat and determining the number of these cells containing more than 10 NK-1R-ir endosomes. Myenteric plexus neuronal cell bodies were identified by their large size, position between the internal and external layers of smooth muscle of the muscularis externa, encapsulation by a perineurium, and their morphology. Cytoplasmic NK-1R-ir endosomes were distinguished from NK-1R-ir plasma membranes or plasma membrane-associated endosomes by ensuring that the nucleus of the neurons was in the same optical section as the NK-1R-ir endosomes.

### 2.9. Statistical Analysis

Results are expressed as mean ± SEM (*N* = 5–15). Mean differences among 2 groups were examined by the Student *t*-test and mean differences among several groups by one-way ANOVA with Dunnett's or Tukey-Kramer posttests, using GraphPad InStat version 3.05 for Windows (GraphPad Software, San Diego, CA). *P* values < 0.05 were considered significant.

## 3. Results

**Figure 1:**
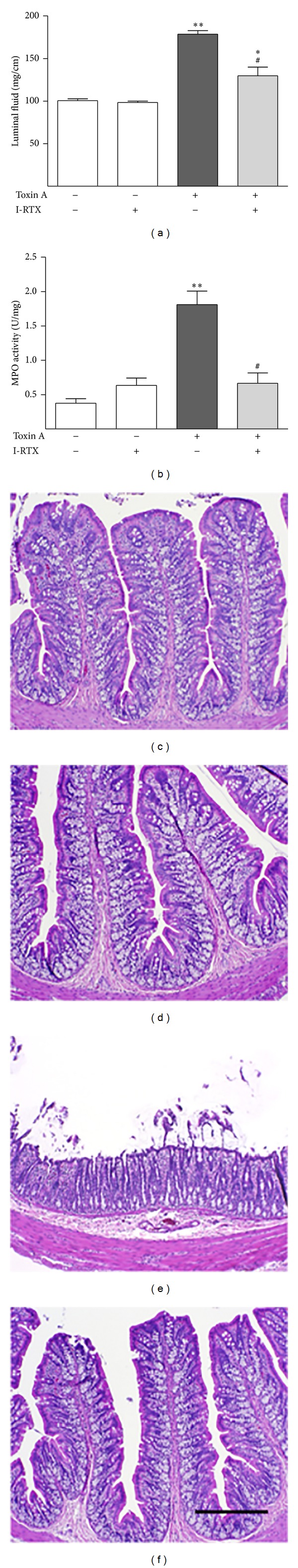
Effects of toxin A on colonic luminal fluid accumulation, MPO activity, and histopathology and inhibition of these effects by TRPV1 antagonism. (a) Toxin A-induced stimulation of colonic luminal fluid accumulation and the inhibition of this effect by pretreatment with 1 *μ*g of I-RTX administered intraluminally 30 min before toxin A. (b) Toxin A-induced stimulation of colonic MPO activity and the inhibition of this effect by pretreatment with 1 *μ*g of I-RTX administered intraluminally 30 min before toxin A. H&E-stained sections of rat colonic segments after they were treated intraluminally for 3 h with vehicle (c), 1 *μ*g of I-RTX (d), 5 *μ*g of toxin A (e), or 5 *μ*g of toxin A after pretreatment with 1 *μ*g of I-RTX 30 min before toxin A administration (f). Scale bar, 100 *μ*m. ∗*P* < 0.05 versus toxin A(−)/I-RTX(−); ∗∗*P* < 0.001 versus toxin A(−)/I-RTX(−); ^#^ *P* < 0.05 versus toxin A(+)/I-RTX(−).

We previously demonstrated that TRPV1 activation plays a role in toxin A-induced inflammation in the rat ileum [14, 15, 20] but the role of TRPV1 has not been examined in toxin A-induced colitis. Therefore, we first sought to determine if pharmacological antagonism of TRPV1 inhibits toxin A colitis as it does toxin A ileitis in rats. Intraluminal administration of toxin A in the rat colon caused an intense inflammation similar to that previously observed in the rat ileum. Toxin A stimulated luminal fluid accumulation, increased MPO activity, and caused intense histopathology characterized by loss of mucosal folding, surface ulceration, and influx of neutrophils (Figure 1). In order to determine if TRPV1 mediates a portion of toxin A-induced colitis as it does ileitis, colonic segments were pretreated for 30 minutes with intraluminal administration of a TRPV1-defunctionalizing concentration of the TRPV1 partial agonist, I-RTX [22], before toxin A injection. I-RTX significantly inhibited toxin A-induced luminal fluid accumulation, MPO activity, and histopathology in the colon (Figure 1) just as it does in the ileum.

**Figure 2:**
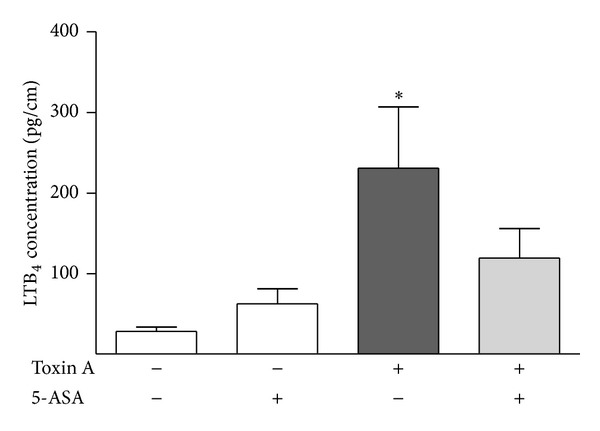
Effects of 5-ASA, toxin A, and toxin A after pretreatment with 5-ASA on colonic tissue LTB_4_ concentrations. ∗*P* < 0.01 versus no treatment.

We previously provided evidence that LTB_4_ mediates the inflammatory effects of toxin A in the rat ileum [15]. Therefore, we examined the effects of toxin A, 5-ASA, and toxin A after 5-ASA pretreatment on levels of LTB_4_ in the colon. Toxin A stimulated a significant increase in colonic tissue concentrations of LTB_4_ (Figure 2). 5-ASA alone had no significant effect on tissue LTB_4_ concentrations. Intraluminal pretreatment of the colonic segment for 30 minutes with 100 *μ*g of 5-ASA inhibited the toxin A-induced increase in colonic LTB_4_ levels (Figure 2).

**Figure 3:**
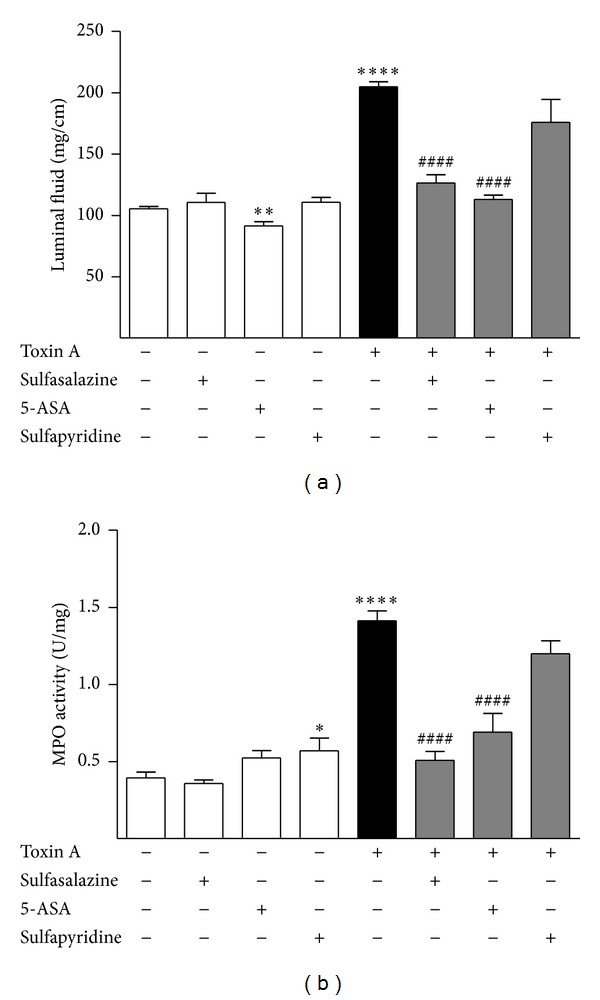
Effects of sulfasalazine, 5-ASA, and sulfapyridine on toxin A-induced colonic luminal fluid accumulation and MPO activity. Sulfasalazine was administered in the drinking water at a dose of 100 mg/kg*·*day for 5 days. 5-ASA and sulfapyridine were administered as pretreatments for 30 minutes before toxin A was injected into isolated colonic segments. (a) Toxin A stimulation of colonic luminal fluid accumulation and the inhibition of this effect by chronic treatment with sulfasalazine or acute treatment with 5-ASA or sulfapyridine. (b) Toxin A stimulation of colonic MPO activity and the inhibition of this effect by chronic treatment with sulfasalazine or acute treatment with 5-ASA or sulfapyridine. ∗*P* < 0.05 versus no treatment; ∗∗*P* < 0.01 versus no treatment; ∗∗∗∗*P* < 0.001 versus no treatment; ^####^ *P* < 0.0001 versus toxin A.

**Figure 4:**
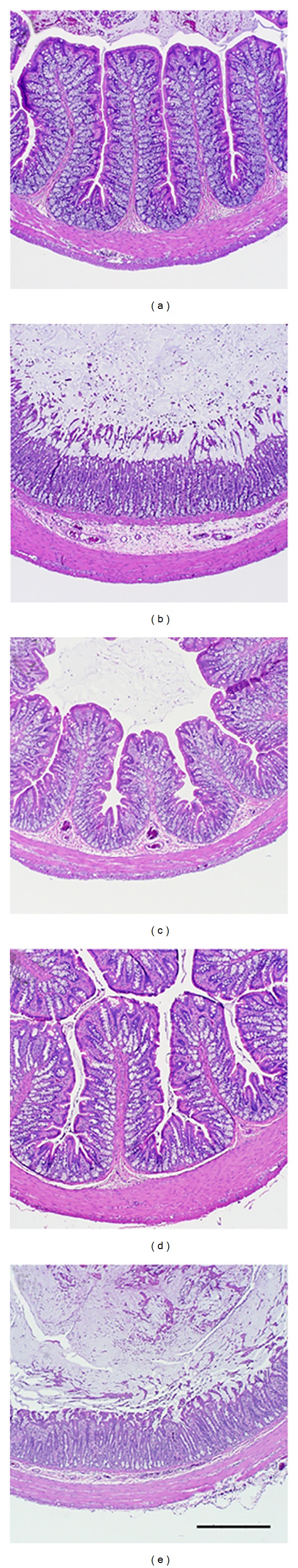
The effects of toxin A on colonic histopathology and protection against these effects by sulfasalazine and 5-ASA but not sulfapyridine. H&E-stained sections of rat colonic segments after they were treated intraluminally for 3 h with vehicle (a), 5 *μ*g of toxin A (b), 5 *μ*g of toxin A after chronic treatment with 100 mg/kg*·*day of sulfasalazine for 5 days (c), 5 *μ*g of toxin A after acute pretreatment with 100 *μ*g of 5-ASA for 30 minutes (d), and 5 *μ*g of toxin A after acute pretreatment with 100 *μ*g of sulfapyridine for 30 minutes (e). Scale bar, 100 *μ*m.

When sulfasalazine was administered chronically to the rats for 5 days in their drinking water, it had no effect by itself but inhibited toxin A-stimulated luminal fluid accumulation and MPO activity (Figure 3). We next tested the effects of the two active constituents of sulfasalazine, 5-ASA and sulfapyridine. 5-ASA given alone caused a small but significant decrease in luminal fluid accumulation but had no effect on MPO activity. Sulfapyridine given alone caused a significant increase in MPO activity but had no effect on luminal fluid accumulation (Figure 3). Pretreatment of the colon for 30 minutes with 5-ASA before toxin A was administered resulted in highly significant inhibition of toxin A-induced luminal fluid accumulation and MPO activity (Figure 3). Pretreatment with sulfapyridine did not inhibit toxin A-induced colonic inflammation (Figure 3). When histopathology was examined, it was clear that both sulfasalazine and 5-ASA were highly protective of the structure of the colon against toxin A inflammation but sulfapyridine was not (Figure 4).

**Figure 5:**
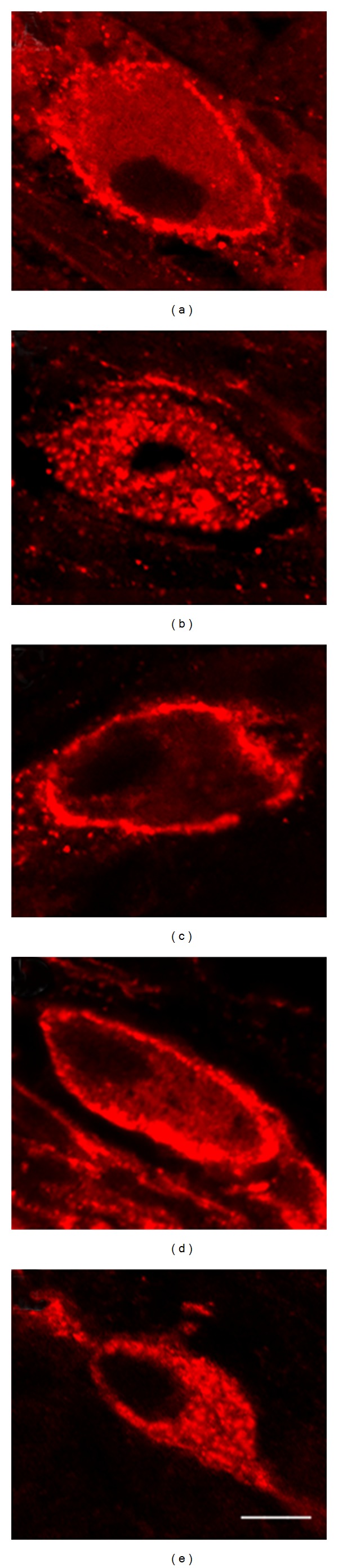
The effects of sulfasalazine, 5-ASA, and sulfapyridine on toxin A-induced substance P release in the colon. Laser scanning confocal fluorescent micrographs of neurons immunostained for the NK-1R from colonic segments treated for 3 h with (a) vehicle, (b) 5 *μ*g toxin A, (c) 5 *μ*g toxin A after 5 days of treatment with sulfasalazine (100 mg/kg*·*day), (d) 5 *μ*g toxin A after 30 minutes of intraluminal pretreatment with 5-ASA (100 *μ*g), and (e) 5 *μ*g toxin A after 30 minutes of intraluminal pretreatment with sulfapyridine (100 *μ*g). Bar, 5 *μ*m.

**Figure 6:**
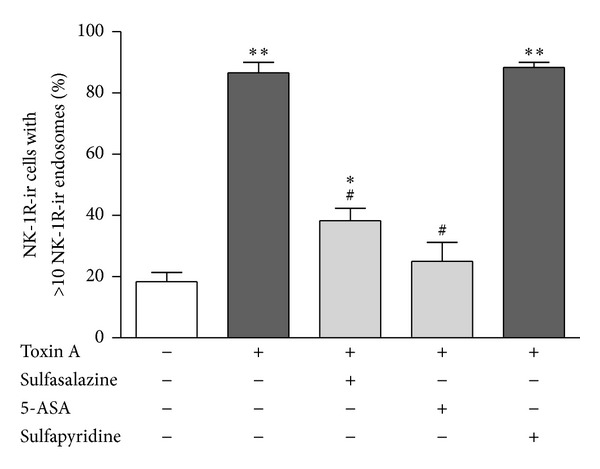
Quantitation of toxin A-induced SP release and its inhibition by sulfasalazine and 5-ASA but not by sulfapyridine. The percentage of NK-1R-ir neuronal cell bodies in the myenteric plexus of the colon with >10 NK-1R-ir endosomes was determined in 20 cells per animal for each treatment as an index of endogenous SP release. Toxin A stimulated SP release and this effect was significantly inhibited by sulfasalazine and 5-ASA but not sulfapyridine. ∗*P* < 0.05 versus no treatment; ∗∗*P* < 0.001 versus no treatment; ^#^ *P* < 0.001 versus toxin A alone treatment.

Because it has previously been shown that toxin A stimulates release of the proinflammatory neurotransmitter SP in the rat ileum via activation of TRPV1 [14, 17], we reasoned that if 5-ASA inhibits toxin A-induced colitis by blocking the generation of an endogenous TRPV1 agonist such as LTB_4_, then 5-ASA and sulfasalazine, but not sulfapyridine, should inhibit toxin A-induced SP release in the colon. Therefore, we measured SP release in response to intraluminal toxin A and tested the effects of 5-ASA on SP release. We used immunocytochemical assessment of NK-1R endocytosis in myenteric plexus neuronal cell bodies as an index of endogenous SP release and as an index of the action of locally released SP as described previously [14, 17, 20, 23]. Intraluminal administration of toxin A caused NK-1R endocytosis, reflecting the action of endogenously released SP, and this effect was inhibited by chronic treatment with sulfasalazine and acute pretreatment with 5-ASA but not sulfapyridine (Figure 5). When quantitated, toxin A-induced SP release was statistically significant and sulfasalazine and 5-ASA significantly inhibited toxin A-induced SP release (Figure 6). Sulfapyridine did not significantly inhibit toxin A-induced SP release (Figure 6).

**Figure 7:**
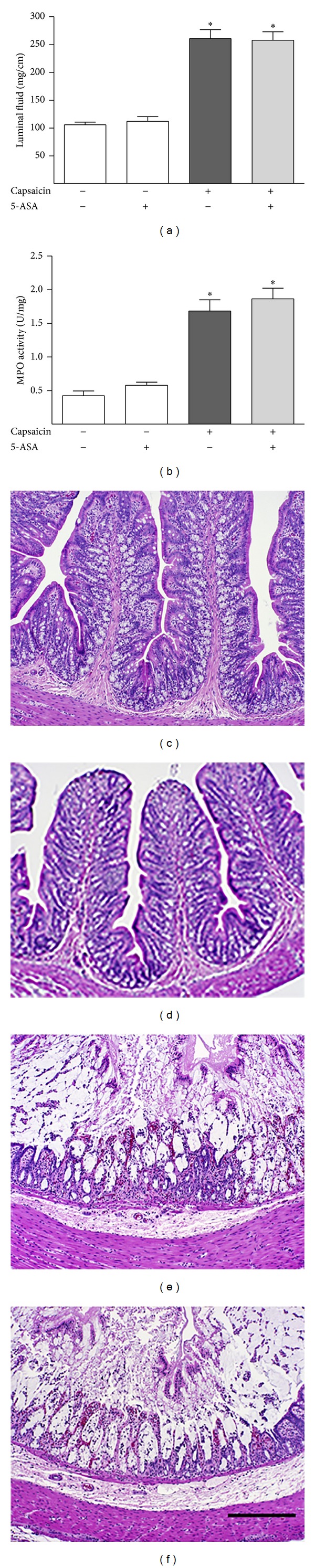
Effects of 5-ASA on capsaicin-induced colonic luminal fluid accumulation and MPO activity. 5-ASA was administered intraluminally at a dose of 100 *μ*g for 30 minutes before capsaicin. (a) Capsaicin stimulation of colonic luminal fluid accumulation and the lack of significant inhibition of this effect by acute pretreatment with 5-ASA. (b) Capsaicin stimulation of colonic MPO activity and the lack of significant inhibition of this effect by acute pretreatment with 5-ASA. H&E-stained sections of rat colonic segments after they were treated intraluminally for 3 h with vehicle (c), 100 *μ*g of 5-ASA (d), 4 mg of capsaicin (e), or 4 mg of capsaicin after pretreatment with 100 *μ*g of 5-ASA 30 min before toxin A administration (f). Scale bar, 100 *μ*m. ∗*P* < 0.001 versus sulfapyridine-/toxin A-.

If 5-ASA blocks toxin A colitis by inhibiting the generation of an endogenous TRPV1 agonist such as LTB_4_, we reasoned that 5-ASA pretreatment should not inhibit colitis caused by an agent that directly stimulates TRPV1. Capsaicin, the pungent ingredient in chili peppers, is an activator of TRPV1 that has been shown to cause enteritis that is very similar to that caused by toxin A in the rat ileum [14]. Intraluminal injection of capsaicin in isolated rat colonic segments caused intense inflammation as assessed by luminal fluid accumulation and increased tissue MPO levels (Figure 7). Pretreatment of the segments for 30 minutes with 5-ASA had no effect on capsaicin-induced colitis, thus supporting the concept that 5-ASA protects against colitis by inhibiting toxin A-induced generation of an endogenous TRPV1 agonist such as LTB_4_. These results also demonstrate that 5-ASA is not itself a TRPV1 antagonist.

## 4. Discussion

Our first goal was to determine if TRPV1 activation plays a role in toxin A-induced colitis as it does in toxin A-induced ileitis in rats [14, 15, 20]. Intraluminal administration of toxin A into isolated segments of rat colon caused an intense inflammation similar to that previously described [24]. When pretreated for 30 minutes with a defunctionalizing dose of the specific TRPV1 partial agonist, I-RTX, toxin A-induced luminal fluid and MPO activity were significantly inhibited and normal colonic mucosal histology was preserved. The anti-inflammatory effects of TRPV1 inhibition in the rat colon are similar to those seen in the rat ileum previously and indicate that TRPV1 activation is important in toxin A colitis as well as in ileitis.

Toxin A also stimulated increased LTB_4_ levels in the rat colon just as it does in the rabbit [25] and rat [15] ileum. We have previously demonstrated that LTB_4_ causes ileitis similar to that seen after toxin A administration and that the inflammatory effects of both LTB_4_ and toxin A are strongly inhibited by TRPV1 antagonism [15]. In addition, pharmacological inhibition of 5-LO activity inhibited both toxin A-induced ileal LTB_4_ production and ileitis [15]. These findings coupled with the observations that LTB_4_ is proinflammatory in human UC and that drugs containing 5-ASA inhibit LTB_4_ production and colonic inflammation in UC led us to hypothesize that 5-ASA may also inhibit toxin A colitis by inhibiting 5-LO in the rat colon. It was previously shown that 5-ASA inhibits toxin A-induced LTB_4_ release and mucosal permeability to mannose in the rabbit ileum but does not inhibit fluid secretion or morphological damage [25]. In the present study, however, 5-ASA significantly inhibited toxin A-induced luminal fluid accumulation and morphological damage as well as MPO activity in the rat colon. These differing results may be due to species differences, organ differences, or other unknown factors.

In order to differentiate the anti-inflammatory mechanism of action of 5-ASA from sulfasalazine, which contains one molecule of 5-ASA coupled to one molecule of sulfapyridine via an azo bond, we separately tested the acute effects of sulfapyridine in the toxin A model of rat colitis. This was important because 5-ASA has been shown to be the active moiety of sulfasalazine in preventing human UC [3] and proctitis [4] and because sulfasalazine but not 5-ASA has been shown to be an inhibitor of the proinflammatory nuclear factor, nuclear factor kappa B (NF-*κ*B) [26]. We found that sulfapyridine had no effect on toxin A colitis in rats, suggesting that 5-ASA is the active component of sulfasalazine in toxin A colitis just as it is in human UC and proctitis.

The present results suggest the possibility that sulfasalazine may be therapeutically useful in treating human* C. difficile* colitis. For example, although this disease is usually effectively treated by antibiotics [27], some cases of recurrent disease have proven difficult to treat [28] and new, more virulent strains of* C. difficile* have emerged [29] that may require new therapeutic approaches.

An interesting observation concerning the present results is that 5-ASA, a drug that has proven efficacious in treating *chronic* intestinal inflammatory diseases such as UC and CD, also strongly inhibits *acute C. difficile* toxin A-induced colitis. Although the causes of UC and CD are unknown, there is strong evidence that they are related to the commensal bacteria of the gut and depend on a T cell-mediated adaptive immune response. In contrast, toxin A colitis occurs in response to bacterial exotoxins, not the* C. difficile* organism itself, occurs too rapidly in the rat model to involve adaptive immune responses, and appears to be mediated largely by a neutrophilic innate immune response [24, 30]. Although it is possible that 5-ASA has different mechanisms of action in inhibiting chronic versus acute intestinal inflammation, it may be that its anti-inflammatory effect is due to inhibition of 5-LO in both cases. Support for this concept comes from animal studies showing that inhibition of 5-LO is also efficacious in blocking chronic colitis resulting from administration of trinitrobenzene sulfonic acid (TNBS) [11, 13, 31, 32]. Additionally, support for the involvement of TRPV1 in chronic colitis comes from animal studies of TNBS colitis [33, 34], dextran sulfate-induced colitis [35, 36], and colitis caused by adoptive transfer of CD4^+^/CD25^−^ T cells in SCID mice [37]. Based on these observations, it will be important in future studies to determine the role of LTB_4_ activation of TRPV1 in the human IBDs.
